# Facilitators and challenges in delivering a peer-support physical activity intervention for older adults: a qualitative study with multiple stakeholders

**DOI:** 10.1186/s12889-020-09990-x

**Published:** 2020-12-12

**Authors:** Anthony Crozier, Lorna Porcellato, Benjamin J. R. Buckley, Paula M. Watson

**Affiliations:** 1 Physical Activity Exchange, Research Institute for Sport and Exercise Sciences, Liverpool John Moores University, Liverpool, UK; 2 Faculty of health, Public Health Institute, Liverpool John Moores University, Liverpool, UK

**Keywords:** Interviews, Focus groups, Barriers, Exercise referral

## Abstract

**Background:**

Involving peer volunteers in intervention delivery can provide social support and improve adherence. Whilst such interventions have the potential to reduce physical activity (PA) intervention costs, little is known about the process of delivering them in practice. This qualitative study explored the facilitators and challenges of delivering a peer-support PA intervention for older adults, with a view to making recommendations for the delivery of future interventions.

**Methods:**

Data were collected via (7) semi-structured interviews and a focus group with stakeholders involved in a peer-support PA intervention for older adults in a large city in the North-West of England. Participants included local authority staff (*n* = 3), peer volunteers (*n* = 2) and service users (*n* = 7). Audio data were transcribed verbatim and thematically coded to identify perceived facilitators and challenges.

**Results:**

Facilitators to delivery included social interaction, community referral pathways, suitable facilities, peer volunteers and high-quality instructors. Challenges surrounded inconsistent practice, staff capacity, safety and accountability, and awareness raising.

**Conclusions:**

Peer volunteers can provide an additional support mechanism alongside qualified instructors for increasing social interaction within PA interventions. For optimal intervention delivery, consideration needs to be given to equipment and space, safety and accountability and consistency of practice.

**Supplementary Information:**

The online version contains supplementary material available at 10.1186/s12889-020-09990-x.

## Background

The benefits of exercise and more generally physical activity (PA) for preventing and treating chronic disease are well established [1, 2]. Subsequently, exercise for health has been legitimised as a welfare policy within the public health agenda, potentially addressing medical and social issues manifested through inactive lifestyles [3]. UK guidance suggests adults (18–64 years) and older adults (65+ years) should aim to be active daily and participate in at least 150 min of moderate-intensity (e.g. brisk walking, cycling), or 75 min of vigorous-intensity (e.g. running) PA per week to gain health benefits [4]. Alongside this, it is recommended that both adults and older adults perform strength and resistance-based activities that focus on working all major muscle groups at least two days a week [4]. Approximately 11.5 million people in the UK are classified as inactive [5] contributing to the increase of non-communicable diseases such as cardiovascular disease, diabetes mellitus, obesity, and ultimately premature death [6, 7]. These figures worsen with age, with around 28% of 55–64-year olds, 31% of 65–74-year olds and 54% of 75+ year olds classified as inactive [5]. For older adults, PA is critical in maintaining and improving fitness, functional mobility, independent living, quality of life and decreasing risk of geriatric-related diseases [8, 9]. Furthermore, PA in older adults has extensive physical (e.g. protective effect on functional decline [10]), mental (e.g. reduced depression and increased self-efficacy [11]) and social benefits (e.g. creation of new social networks and reduced loneliness [12]).

Although the benefits of PA for adults are well documented, substantial barriers exist to the uptake and long-term adherence of PA [13]. Commonly reported barriers include time, costs, accessibility, and lack of motivation [13]. Older adults face additional barriers such as medical conditions, fear of injury, social influence, lack of energy, lack of support and environmental concerns [14]. Evidence from both qualitative and quantitative international studies has shown peer-support interventions to be effective at increasing older adults` PA levels [15, 16], during which peers (current or previous programme attendees) provide moral support for participants [17–19]. Social support itself, has been identified as a key requirement in engaging older adults in PA [20]. Peer involvement during community-based PA interventions can increase retention and adherence [21], primarily through increased social interaction and relatedness within sessions [15–17]. Previous research [22] shows the adoption of a motivational role by peer support workers improves self-efficacy, perceptions of confidence and self-determination within participants. Face-to-face and two-way communication between peers and service users has led to mutually positive relationships through increased honestly, empathy and transparency [23, 24].

The peer support role has been developed further in some interventions into peer coaching [23, 25]. Unlike peer support where a qualified member of staff leads all aspects of the session, a peer coaching model allows less input by instructors (sometimes none), and has therefore been recognised as filling the void for intervention delivery and as a tool for sustainable interventions [23]. Peer coaching has been shown to produce comparable PA intervention outcomes to professional delivery, suggesting peer coaching within interventions has potential to reduce implementation costs and increase delivery capacity [16, 23, 25]. Vijver et al. [25], however, identify that in the short-term health professionals may need to set-up and lead such interventions as they are acknowledged as being skilled in this field, with peer coaches being able to learn from them and in the future continue with limited professional support once they become more experienced. In this sense, there may be a developmental transition from peer support to peer coaching. Notably, despite differences in the levels of responsibility afforded, both peer support and peer coaching share a focus on motivational and relational support from peers (often provided on a voluntary basis).

Further research is required to understand the facilitators and challenges of involving peers to assist with intervention delivery [18]. Ginis et al. [17] identify that more practice-based evidence is required concerning the factors that facilitate peer-related interventions, including “what works, where and why?”. Previous studies [18] have also identified that outcomes for both peer volunteers and intervention participants need further development to ensure that a positive experience is being had. The development of peer volunteer roles has been discussed in previous studies [24–26]. How this is achieved requires further exploration, specifically their involvement within sessions and how this can be expanded from their perspective [24]. Such process information is important in aiding future replication and enhancing sustainability of future interventions [27].

The aim of this study was to conduct a qualitative process evaluation of an established peer-support PA intervention for older adults in a large city in the North-West of England. The intervention was run by a local authority in a single leisure centre and involved group-based sessions, primarily targeted at over 50-year olds, but retaining open access for older adults if referred by health care professionals (HCPs). The intervention was supported by peer volunteers, who assisted the exercise instructor in performance monitoring, encouraging others and providing social support during and after sessions. Following promising retention figures policymakers were interested in replicating the intervention across the city and required a better understanding of the factors important for successful delivery. This study therefore aimed to explore the facilitators and challenges of delivering a peer-support PA intervention for older adults, with a view to making recommendations for the delivery of future interventions.

## Method

### Design

Multiple qualitative methods (semi-structured interviews, focus groups) were employed to enable a deep exploration of the subject matter both individually and collectively, and to provide an insight into multiple stakeholder perspectives over a similar timeframe. Participants were unpredictable in their attendance which made it difficult to just use one singular method to obtain a sufficient cross-section of participant feedback. Therefore, interviews were used to capture the experiences of individuals who attended different sessions within the intervention and a focus group was used to encourage peer interaction and promote shared experiences from individuals within the same (most popular) session (e.g. [28]). Very few participants had a relationship prior to attending the intervention, with the majority forged through interactions on site. Research team involvement was limited to participant recruitment and data collection, and the researcher had no prior relationship with the intervention or participants.

### Participants

All staff involved in running the intervention (*n* = 4), current peer volunteers (*n* = 12) and service users (*n* = ~ 50) were invited to take part in the study by the first author. Recruitment was based on convenience sampling. The first author gave a verbal announcement asking if attendees wanted to participate in the study once they had finished a session. If attendees were interested, they were provided with a written study information sheet and a day / time was arranged for an interview or the focus group attendance. No one formally declined or stated any reasons for not taking part, but often participants cited nervousness at being interviewed so did not volunteer. The final sample included (3) staff members (centre manager who oversaw the intervention, the exercise referral instructor who delivered the sessions and a health trainer who worked in the community and signposted people to the sessions for health benefits), (2) peer volunteers and (7) service users (1 male). All staff members were White British, female (mean age 42 years) and employed full-time by the local authority. Peer volunteers were both White British, male, retired (mean age 66 years), had various long-term medical conditions and lived within 3.5 miles of the intervention. Service users taking part were all White British, retired, had various long-term medical conditions, and had a mean age of 63 years. Written informed consent was gained prior to study commencement and a reimbursement for participant was provided to each service user and peer volunteer of a £10 shopping voucher. Given the small sample size, it is important to note that “data saturation” or “data adequacy” could be validated as no new themes were identified when analysing the final few transcripts, an area previously discussed by Hennink, Kaiser & Weber [29] and Braun & Clarke [30].

### Peer-support PA intervention

The peer-support PA intervention began in 2016 and was established via a partnership between the local authority, local clinical commissioning group and strategic stakeholders. The intervention was aimed at increasing PA levels of older adults through a subsidised 12-week programme, including gym-based classes and a range of physical activities (e.g. walking football, walking netball, bowls, swimming, aqua fit), followed by ongoing discounted access to the leisure centre. Full details of the intervention are provided in Table 1.

**Table 1:** Intervention components mapped onto items 1 to 9 of the TIDieR Checklist [31]

Item Number	Item
**Brief Name**	
**1.**	Peer-support PA intervention for older adults
**Why?**	
**2.**	Peer-support intervention that aimed to increase older adults` PA levels. Peer support was provided during the sessions in the form of motivation and assistance with activities when required. This form of intervention has previously been recognised to increase adherence to PA [15–17]. Attendance data showed that 93% of attendees were retired, 76% were female from a white ethnic background and 26% lived > 3.6 miles from the venue.
**What?**	
**3.**	**Intervention resources**
	Consultation paperwork – A physical activity readiness questionnaire (PARQ) was completed to ascertain if the service user had any medical conditions or medications that the instructor should be aware of. Once completed, a brief conversation about service user goals and service provision was undertaken where possible with the exercise referral instructor.
	Specialised, adapted gym equipment (hydraulic / air resistance controlled) was available for gym-based sessions in a specific lower-gym room that was only accessible to intervention users. All users also had access to a main sports hall where equipment was available for activities such as netball, football, or bowls. The swimming pool was also available for swimming or aqua sessions.
**4.**	**Procedures and key components**
	The intervention adhered to the National Institute for Health and Care Excellence (NICE) guidance for exercise referral [32] which advocates a focus on inactive individuals and ongoing support, with self-referral and health care professional (HCP) referrals accepted.
	**Eligibility:** Open access to all ages, but targeted individuals aged over 50 years. Medical conditions were allowed, but not essential for access.
	**Week 1 Consultation (induction):** Introduction to the fitness centre, health screening and peer-support sessions available conducted by the exercise referral instructor. Mainstream gym induction (where possible) also completed by the exercise referral instructor so that service users could attend the main gym in addition to the intervention sessions in the lower gym. 12-week subsided access to peer-support classes, swimming and main-gym commenced after the induction.
	**Week 12:** At the end of the subsidised period, service users were able to continue attending the sessions (gym-based classes, walking football, walking netball, bowls, swimming and aqua fit) at the centre, but were now required to either pay a non-subsidised entry fee per session or join on the off-peak membership offered by the fitness centre.
**Who will provide?**	
**5.**	**Referring health professionals:** Referrals were accepted from all health professionals (GP’s, physiotherapists, nurses, etc.) but data showed that GPs were the chief source of referrals (31%), followed by health trainers (17%).
	**Exercise referral practitioners:** Based at local authority fitness centres they were qualified to lead the sessions with support of the peer volunteers.
	**Health trainers:** Local authority staff who worked in the community and signposted the general public to health-related services. Health Trainers were already trained to provide lifestyle advice for numerous health-related behaviours (physical activity, weight loss, smoking cessation, nutrition etc.) and could signpost service users to the peer-support intervention.
	**Peer volunteers:** People who had (usually) attended the peer-support intervention themselves (some remained service users) and had given up their time to assist in the running of the sessions. They set up sessions by moving equipment, monitored the timings throughout sessions, motivated service users, increased interaction levels with service users and hosted external events such as raffles, Christmas parties, etc. Peer volunteers had no formal qualifications and could not lead sessions independently, therefore a staff member was always present due to insurance guidelines.
	**Centre manager:** Oversaw session provision by ensuring staff were present. Ensured all peer volunteers were registered with the local authority and provided a suitable venue space and equipment for sessions to take place.
**How?**	
**6.**	**Peer-support sessions:** Provided in face-to-face format to small groups of service users (8–15 participants) by the exercise referral instructor.
**Where?**	
**7.**	All service user consultations and activities took place at the local leisure centre.
**When and how much?**	
**8.**	The peer-support intervention had an initial 12-week subsidised period and supported older adults to increase their PA levels. The programme consisted of daytime (9 am – 3 pm) group-based sessions such as circuits, walking football, walking netball, indoor bowls, aqua and chair-based exercise which were primarily led by a qualified exercise referral instructor and supported by peer volunteers. Swimming could also be completed in designated sessions without specialist supervision. Each session lasted approximately 45 min to 1 h.
**Tailoring**	
**9.**	The peer-support sessions were group-based but recommended to service users based on individualised needs alongside their PA preferences. All sessions were aimed at service users aged 50 or over.

### Semi-structured interviews

As this was a pragmatic study born out of a local public health concern, the semi-structured interview guide (see additional file 1) was developed based on the questions deemed most pertinent to meet local public health needs (e.g. what factors were important for the intervention’s success, what challenges did stakeholders face, and what areas needed improvement?). Within this, we maintained a broad focus and used open questions to allow stakeholders to respond with the issues they deemed most important, since previous research has shown factors that influence older adults’ PA participation can occur on a number of individual, social and environmental levels (e.g. [33]). Interviews (*n* = 7) were conducted on an individual basis by the first author (alone) with (3) local authority staff, (2) peer volunteers and (2) service users. Interviews took place in a private office space within the leisure centre, lasting 28 min on average (ranging from 10 min to 53 min). Pilot interviews were conducted by the first author with three independent researcher peers prior to study commencement to enhance credibility and refine interview questions where necessary [34]. Prompts and probes were used during interviews to elicit more detailed responses from participants where appropriate [35]. At the end of each interview, a brief verbal summary was provided by the researcher to clarify the main points and allow participants to add further information [36].

### Focus group

One focus group (with different participants to the semi-structured interviews) was conducted by the first author with service users (*n* = 5) who had been through the 12-week intervention in a private conference room at the leisure centre. The focus group lasted 39 min and was based on the same semi-structured interview guide used in the interviews. Spontaneous conversation was encouraged by the facilitator with the peers able to discuss and challenge opinions if they wished [37]. Participants were familiar with each other meaning the interaction incorporated real-life recollections whereby agreement and contradictions added to discussion topics [37]. Clarification of information was sought during the questioning process to ensure participants were able to expand on each-others opinion and summarise the information provided [37, 38].

### Data analysis

Data obtained through the focus group and semi-structured interviews were audio recorded using a portable Dictaphone and transcribed verbatim. Interview transcripts were thematically analysed manually via a six-phase process as recommended by Braun and Clarke [39]: data familiarisation, generating codes, searching for themes, reviewing themes (narrowing in relation to facilitators and challenges), defining and naming themes, and writing up. Themes were generated based on patterns of response and relevance to the pre-determined categories formed by the research questions [39]. Flexibility in analysis was driven by both the prevalence (number of speakers articulating the theme) and the importance placed on information [39]. This method of coding allows all interview information to be thematic with focal points for discussion and future recommendations acknowledged [40]. Primary analysis was conducted by the first author with frequent debriefing sessions with the research team to discuss, challenge and reframe the thematic structure [40].

### Ethical approval

Ethical approval was obtained from the local institutional ethics committee in May 2018 [ref: 18/PHI/020].

## Results

Interview and focus group data were combined to provide a multi-stakeholder insight into the facilitators and challenges identified in delivering peer-support interventions. Table 2 illustrates the themes and subthemes identified during the analysis supported by verbatim quotes. There were five themes for facilitators; social interaction; community referral pathways; facilities; peer volunteers; high quality instructors. There were three themes for challenges; inconsistent practice; capacity, safety, and accountability; raising awareness.

**Table 2:** Facilitators and challenges within the FA intervention

Facilitators / Challenges	Themes / Sub-themes	Illustrative quotes
**Facilitators**	**Social interaction**	I think that the social interaction reallyplays a major part in retention, in that age group anyway (Staff 2)
	**Community referral pathways**	The health trainers often refer people to the intervention (Staff 2)
	**Facilities**	The facilities here, unbelievable We need support and training, butlet’s be honest, without the building we couldn’t do it (Peer volunteer 1)
	**High quality instructors**	I think that what’s made it a success is that we’ve been given good friendly instructors that are easy to get on with and are part of creating a good atmosphere (Service user 7)
**Challenges**	**Inconsistent practice**	
	**-Eligibility**	**View 1:** From what I know it’s over 50 50’s, so under 50 you can’t (attend) (Staff 3)
		**View 2:** We accept all ages, but it’s got to be an acceptable age, probably 30 or 40 (Staff 1)
	**-Induction protocols**	**View 1:** They don’t have to have one, [induction] no. If it’s just the class they want to use they don’t have an induction (staff 2)
	**Capacity, safety, and accountability**	We knew we needed more numbers (staff), one volunteer can only look after three people in the gym (Peer volunteer 2)
	**Raising awareness**	more work needs doing to make GPs aware (Staff 2)

Overall, the peer-support intervention was acknowledged as beneficial for participant health: “I suffer from depression and yet I come in here, it does help me” (Service user 1). Staff also recognised that retention levels were high: “it is a very successful scheme we do get most people that come through [the intervention] actually staying” (Staff 2). Service users reported how the intervention assisted them in meeting new people and enhanced their support networks.

### Facilitators

#### Social interaction

All stakeholders recognised that the social benefits of the intervention were a major factor in both the uptake and adherence levels for participants. It was evident that the group-based sessions had a positive impact on increasing social engagement of service users who may have previously been struggling to develop support networks or retained a level of social isolation or loneliness:

*… I can see who's matching up with who, and then all of a sudden, they're friends, and they go to the same sessions … It's more sociable because it's not one-on-one, everyone just comes for a giggle as well as an exercise.* (Staff 1)

Loneliness itself was discussed by multiple service users: “it [the intervention] keeps people from sitting in the house (service user 1) and “people attend because they are lonely, it [the intervention] gives them something to do” (service user 2). Such social benefits were identified as being fostered by the peer volunteers due to the motivational positions they held which created a positive atmosphere during sessions. All stakeholders consistently acknowledged participant and peer volunteer discussions about previous shared or ongoing experiences of the programme led to a community feel, whereby the physical benefits, although important, were less of a driving force for adherence.

#### Community referral pathways

Service users reported that health trainer and community-based HCP referrals (signposting of individuals in the community to health-related interventions) in the city appeared to be increasing. This enhanced working relationship was demonstrated by the improvement in the referral pathway between the health trainer, local HCPs, and intervention uptake. Service users acknowledged that they were signposted into the service via this route, while both staff and peer volunteers acknowledged that this route was becoming more viable:

*… (we have a) very proactive health trainer at our leisure centre. We work very, very closely and obviously, because their role is to introduce people that would not normally come into a sports centre, and in some respects, to be their buddies, and then they normally pass them over to* [the intervention]. (Staff 2)

#### Facilities

Stakeholders recognised that facilities played a vital part in the delivery of the peer-support intervention due to the quiet nature of the facility (when compared with other leisure centres in the city that were perceived to be more “commercial”). Where other sites often experienced logistical difficulties such as a lack of dedicated space or equipment, the facility where the intervention was running was perceived as optimal for the delivery of an intervention for older adults: “we’ve got exclusive use of a fitness area, also the space and the equipment because they’re not using your everyday gym equipment, they’re using the Matrix stand and click system one to six [hydraulic / air resistance controlled]” (Staff 1).

#### Peer volunteers

Service users highlighted the importance of the peer volunteers in allowing the intervention to run due to the high number of attendees per session: “without the volunteers it’d be hard because you’re getting fifteen, sometimes twenty to a class, and there’s two rooms” (Peer volunteer 1). The peer-support element of the intervention was also perceived to contribute to increased engagement:

*… there are three or four volunteers on site within gym circuits, they're allowed to get involved by sort of assisting with the structure of those exercises … people want to be with their own peers. They have the opportunity of having an exclusive session, they're with the same age group, they become friendly.* (Staff 2)

#### High quality instructors

The provision of highly qualified, friendly staff with the knowledge and experience required to deliver the sessions was recognised as vital (due to the risks involved in group exercise with older adults, who often have multiple co-morbidities). Such competence created confidence on behalf of the service users and HCPs in the service they were accessing / referring to, respectively: “well the instructors are the main ones because when you come onto the scheme, they can tell you exactly what you are capable of or not” (Service user 1).

### Challenges

#### Inconsistent practice

Although eligibility criteria were established and protocols for enrolment on the intervention were in place (see Table 1), staff interview responses suggested there was inconsistency in how these protocols were applied in practice.

#### –eligibility

One of the challenges identified related to the target age and eligibility criteria for the peer-support intervention. Staff responses were inconsistent and suggested a discrepancy between the intended target audience and the inclusion criteria that was applied in practice. All staff noted that attendees were generally over the age of 50 years. However, some staff were under the impression that only those 50 years and upwards could access the peer-support intervention, whilst other staff believed the intervention was open to all adults:

*… from what I know, it's the over fifties.* (Staff 3)

*… Some people say over fifty, but why not anybody?* (Staff 1)

*… we accept all ages … It's got to be the acceptable age, so it's probably thirty, forty … we do not want to make age a barrier … but its predominantly 50-plus* (Staff 2)

#### – induction protocols

Staff perspectives also highlighted discrepancies in the way the induction process was approached. Some staff noted that mainstream gym inductions were a necessity for all intervention attendees. In practice however, it appeared that service users only attended a gym induction if they wanted to use that area of the building and staff reports suggested many service users did not receive an “official” consultation when starting the intervention (other than completing a Physical Activity Readiness Questionnaire (PARQ) and a brief introduction to session activities). This inconsistency in induction processes was particularly evident when participants were referred via a health trainer; participants were invited to a meet and greet with the health trainer and then if they wished they could then join in the peer-support sessions, without any formal induction meaning that health status could go unmonitored and class suitability not checked.

#### Capacity, safety, and accountability

There was a constant acknowledgement that instructor and volunteer availability were key components for intervention success, and therefore, recognition that maintaining safety during holiday periods or times of sickness was difficult. Such issues could lead to sessions being cancelled, delivered by less experienced instructors or running with less than optimal levels of volunteers: “I’m not that brave to cancel those sessions … by hook or by crook I have to move somebody who’s a qualified instructor, to go up there and take those sessions” (Staff 2). A further challenge was that sessions were scheduled during the daytime when the facility was quieter, which in turn restricted access for participants who worked. This did however sometimes lead to participants choosing to join the gym instead: “people who I think struggle with times tend to come out of [the intervention] and want to join as a gym member” (Staff 3).

#### Raising awareness

All stakeholders indicted that awareness of the peer-support intervention needed to be improved among HCPs, specifically GPs:

*… I don’t think a lot of people know about it.* (Peer volunteer 1)

*… numbers just weren't coming in, the material was out, you know, leaflets … It's just that link.* (Staff 3)

## Discussion

This study explored the facilitators and challenges of delivering a peer-support PA intervention for older adults, drawing on views of multiple stakeholders. There was broad agreement between staff, peer volunteers and service users that interpersonal factors (i.e. social interaction, peer volunteers, high quality instructors) were important in enhancing intervention engagement. Other facilitators included community referral pathways and facilities, which featured within all of the stakeholder discussions. Challenges included inconsistent practice (eligibility and induction procedures), capacity, safety and accountability and the need to raise awareness of the intervention amongst HCPs.

Participants in our study noted peer involvement had a positive influence on PA adherence. Peer volunteers were deemed to be important motivators and enhanced single instructor-led sessions by providing additional support (as also noted elsewhere [15–19, 21]). One reason for peer-support implementation is that attendees can develop a positive shared experience with their peers, staff, and volunteers [18, 21, 23, 24]. Participants in the current study noted that high levels of optimism, compassion and the friendly nature shown by both the peer-support volunteers and the qualified instructor fostered increased interaction and social inclusion across the groups [41]. Such characteristics have been shown in previous studies to enhance participant confidence and incentive to attend [21, 42]. This additional support was however only of benefit when the qualified instructor was present, since the (unqualified) peer volunteers were not able to lead sessions alone or take accountability for the health and safety of participants. This in turn meant there was no cover for instructor holiday or sickness and placed demand on the local authority to increase numbers of qualified staff to maintain the programme. This highlights an important distinction between interventions that are “peer-supported” (e.g. the current study) or interventions that are “peer-coached” or “peer-led” (e.g. Ginis et al. [17]), with the latter requiring elements of training and accountability protocols to be in place to allow peers to lead sessions under minimal (if any) supervision. This also raises the question of whether peer coaching (non-professional coaching provided by peers with shared characteristics), identified by Vijver [23, 25], is feasible in either local authority run facilities or in older populations who may have long-term medical conditions. One solution to this challenge, which was suggested by peer volunteers, would be investment into volunteer training programmes to enhance and possibly expand the current service in keeping with previous interventions [17]. The necessary skills within the training would not just focus on exercise delivery, but also the “soft” skills needed to engage with older adults in this setting (e.g. empathy and compassion [41]). There would, however, be cost implications for the local authority if this were to happen and as highlighted by Vijver [23, 25], experience of delivering interventions would also be a key requirement in maintaining service user trust.

High quality instructors (identified by both skillset and demeanour) and a quiet facility were identified as facilitators of intervention delivery, with specific staff qualifications, suitable equipment and relevant space seen as necessities. Waters et al. [43] however recognised that any community setting should be suitable for PA interventions for older adults assuming that relevant risk assessments are completed. Therefore, although the quiet, well-equipped space was perceived by participants to be bespoke to the local leisure centre, it is not to say that community expansion into different venues on a wider scale would not be possible (with relevant planning and discussion) [43]. Additionally, community referral pathways were recognised as having a positive effect on intervention uptake, with health trainer involvement bridging the gap with HCPs in keeping with a holistic social prescribing landscape [44]. Social prescribing relies upon appropriate community structures (e.g. third sector organisations, community groups and voluntary services) to be in place to support referrals and has previously been used to facilitate PA interventions [42]. In the current study however, there was perceived to be a lack of awareness of the peer-support intervention across local HCPs, something which has previously been recognised as a challenge in PA interventions [45].

A final challenge related to inconsistent practice surrounding eligibility and inductions. Whilst eligibility criteria were in place, the way these were interpreted by staff varied. Additionally, not all service users received a formal induction as recommended by the American College of Sports Medicine (ACSM) [46], which suggested the intervention was not being delivered as intended. The lack of a formal induction was most notable when service users were referred by a health trainer, which was more akin to a social prescribing framework [44]. Missing this formal 1–2-1 with the instructor could increase risks associated with health conditions and reduce goal / behaviour change attainment [47, 48]. These inconsistencies occurred despite standardised protocols being in place regarding eligibility and enrolment on the intervention (which were aligned with current recommendations for older adults) [47]. Our data do not allow us to elucidate the reasons for these inconsistencies, but it is plausible that the busy leisure centre environment in which the majority of working hours were “customer-facing” left little time for reflection of shared working practices. Furthermore, leisure centre staff often have limited access to e-mail or online forms of communication, yielding circulation of group messages a challenge [49]. Whilst standardised protocols are helpful in ensuring consistent delivery, existence of protocols alone does not always lead to uniform practice [50] and additional efforts (such as regular staff meetings) may be required to facilitate the implementation of these protocols in practice.

### Strengths and limitations

Strengths of this study related to the exploration of a peer-support intervention for older adults and the inclusion of multiple stakeholders in the process, both of which are limited across the PA evidence base [17, 18]. The inclusion of various stakeholders allowed a greater understanding of facilitators and challenges from participants with different perspectives and outcome objectives, which can then inform more meaningful interventions in the long-term. As several of the facilitators and challenges raised were not specific to peer-support interventions, our results may contribute to the understanding of implementation factors for PA delivery more broadly. The study was however limited by a small, convenience sample from one local authority site, therefore some caution must be taken in generalising to the wider population.

### Recommendations for practice

Further exploration into PA interventions that include both peer volunteers and qualified instructors working side by side is important to improve understanding of the enhanced support mechanisms and engagement forged through this design. Similarly, risk assessing the environment and utilising suitable space and equipment was identified as important when delivering PA to older adults. Although the peer-support intervention in this study had these elements in place within a leisure centre, future practice should consider wider environments within the community that could be adapted to support such interventions and expand links with health trainer and social prescribing pathways. Additionally, consistency is needed in terms of eligibility and induction procedures, even if the intervention is different depending on service user needs.

## Conclusion

The aim of this study was to explore the facilitators and challenges of delivering an established peer-support PA intervention for older adults in a large city in the North-West of England. This study provides an insight into some of the pragmatic factors that need to be considered for peer-support interventions to work in practice. A major facilitator was the high level of social engagement forged within sessions, primarily through interaction with service users by peer volunteers and qualified instructors. Subsequently, high levels of peer relatedness between the service users and peer volunteers may have resulted in improved adherence to the intervention sessions.

Additionally, findings highlighted the importance of distinguishing between peer-led, peer coached and peer-support interventions, and the different requirements for accountability that come with this, i.e. for peer support there is the advantage of not needing loads of training/qualifications to assist in the intervention etc., but on the downside this means peers cannot reduce the “burden” on the qualified exercise instructor … so in this sense do not increase the delivery capacity of interventions. Further challenges were also noted relating inconsistent practices amongst staff during the eligibility process. Future research should explore how to establish effective processes for involving peer volunteers whilst maintaining the social benefits and potentially increasing delivery capacity in an affordable way.

## Supplementary Information

**Additional file 1.** Interview guides cited in text are provided for service user, peer volunteers and staff.

## Data Availability

The datasets used and / or analysed during the current study are available from the corresponding author on reasonable request.

## Abbreviations

PA: Physical Activity
HCP: Health care professional
GP: General practitioner
NICE: National institute for health and care excellence
NHS: National Health Service
PARQ: Physical activity readiness questionnaire
