# Supplementary material for: Facilitators and challenges in delivering a peer-support physical activity intervention for older adults: a qualitative study with multiple stakeholders
Source: BMC Public Health. 2020 Dec 12;20:1904. doi: 10.1186/s12889-020-09990-x (PMC7733256; doi:10.1186/s12889-020-09990-x)
Supplement: Supplementary file 1 — Additional file 1. Interview guides cited in text are provided for service user, peer volunteers and staff. [file 12889_2020_9990_MOESM1_ESM.doc]

**Interview guide**

**Introduction**

The interview will last 15-45 minutes and will explore 3 main areas; how you / people get referred to the intervention, what works well and what the challenges are.

1. **Service User interviews**

**To begin, could you talk me through how you came to access this physical activity intervention?**

***Prompts & Probes**

- How did you hear about the intervention?
- How did you get access?
- What motivated you to visit your health professional (if applicable) or who suggested that attending the intervention would be good for you?
- Did you attend a consultation prior to, or during the intervention, if so:
  - When was this?
  - What did this include?
  - Who conducted it?
- Had you heard of the intervention prior to you attending? If so,
  - What information did you have and where did you get it?
  - Had you thought about attending the intervention previously?

**Now I’d like to talk about what you think works well (facilitators) in the intervention.**

***Prompts & Probes**

- What works well at the intervention? Expand on if mentioned:
  - Staff
  - Equipment
  - Facility
  - Peer-support
  - Volunteer roles?
- What do you enjoy about the programme?
- What reasons are there for you to keep coming?
- Have you found any benefits from attending, if so, consider:
  - Physical
  - Mental
  - Social
- How accessible is it for you to attend with regards to times / location?
- What was it that motivated you the most to attend the intervention?

**Now I’d like to talk about what you think the challenges are in providing the intervention.**

* **Prompts & Probes**

- What do you think the challenges are in running this intervention? Expand if mentioned:
  - Who looks after you during the sessions?
  - What do the peer volunteers do during the sessions?
  - Where do the sessions take place?
  - How did you find the induction process?
  - Do you know how else you can access this intervention?
- Are there any areas of the intervention that you think would be hard to keep providing?

**(If applicable and not already done this) Finally, could I ask if you see yourself continuing after the 12 weeks?**

***Prompts & Probes**

- What is it that makes you feel this way?
- What do you think the retention levels are – have your friends continued?

Thank you, that’s the end of my questions. To finish off, could I summarise my understanding of your initial access to the intervention, the main things that work well (facilitators) and the main challenges you encountered (summarise key points)…..have I understood your views correctly or is there anything you would like to add?

1. **Peer volunteer interviews**

**To begin, could you talk me through how people come to access this physical activity intervention?**

***Prompts & Probes**

- Did you attend the intervention prior to becoming a volunteer? If so, consider:
  - Why did you choose to attend?
  - How did you find out about it?
  - How long did you attend?
  - What did you do once the 12 weeks had finished?
  - Where their volunteers when you attended?
  - What did the volunteers do?
- How long have you been a peer volunteer? Consider:
  - What is your role within the intervention?
  - How do you assist?
  - How often do you assist, e.g. days / hours?
  - Do you do anything else other than assist in the sessions as a peer volunteer?
- What made you want to be a volunteer within the intervention?
- How do people hear about the intervention?
- How do they get access?
- Which health professionals can refer into the intervention? Leading to - Who do you think refer the most?
- Do participants have to attend a consultation prior to, or during the intervention, if so:
  - What does it involve?
  - Who conducted it?

**Now I’d like to talk about what you think works well (facilitators) in the intervention.**

***Prompts & Probes**

- What works well at the intervention? Expand on if mentioned:
  - Staff
  - Equipment
  - Facility
  - Peer-support
  - Volunteer roles
- What do you enjoy about the programme from a volunteer perspective?
- What reasons are there for you to keep coming and being a volunteer?
- Have you found any benefits from attending, if so, consider:
  - Physical
  - Mental
  - Social
- Is there anything that could improve the intervention?
- Is there anything the intervention could do to make your role easier, or potentially expand it for the better?
- Do you think this intervention could be used across other sites? If so, consider:
  - What would need to be put in place?
  - Would volunteers be needed? If so, why?

**Now I’d like to talk about the challenges are in providing the intervention.**

* **Prompts & Probes**

- What are the challenges are in running this intervention from a peer volunteer perspective? Expand if mentioned:
  - Staffing the sessions?
  - Peer volunteer responsibilities during the sessions?
  - Where do the sessions take place?
  - Holidays / sickness cover
  - Equipment
  - Timings of sessions
  - Number of volunteers
  - Qualifications of volunteers
- Are there any areas of the intervention that you think would be hard to keep providing if there were no volunteers?
- What do you think the retention levels are –have people you have met often continued?

1. **Staff interviews**

**To begin, could you talk me through how a service user can access this physical activity intervention?**

***Prompts & Probes**

- How do they hear about the intervention?
- How did they get access?
- Which health professionals refer? Who refers the most (category, e.g. GP?)
- Who is the intervention for? Consider:
  - Age restrictions?
  - Locality?
  - Medical condition requirement?
  - What format?
- Do they attend a consultation prior to, or during the intervention, if so:
  - When is this?
  - What does this include?
  - Who conducts it?
- Is the intervention actively promoted? If so,
  - What information is available?
  - Where can you get it?
  - What format?
- What PA opportunities are there in the intervention?

**Now I’d like to talk about what you think works well (facilitators) in the intervention.**

***Prompts & Probes**

- What works well at the intervention? Expand on if mentioned:
  - Staff
  - Equipment
  - Facility
  - Peer-support
  - Volunteer roles
- What do you think participants enjoy most about the programme?
- Is there a good retention level (in your opinion)?
- What reasons do you think that they keep coming?
- Have you found that participants mention any benefits from attending, if so, consider:
  - Physical
  - Mental
  - Social
- How accessible is it for participants to attend with regards to times / location?
- Can you think of any other things that work well that we have not mentioned from a staff perspective?

**Now I’d like to talk about what you think the challenges are in providing the intervention.**

* **Prompts & Probes**

- What do you think the challenges are in running this intervention? Expand if mentioned:
  - Staff levels
  - Promotion
  - Funding
  - Peer volunteer recruitment
  - Consultation process
  - Referring professional issues
- Are there any areas of the intervention that you think would be hard to keep providing?
- Could this intervention be transferred to other sites? Please expand?
- Are there any other challenges that we have not mentioned from a staff perspective?

**If no**

Thank and finish interview.

*Prompts/Probes are there to be used as a guide for the interviewer. They are flexible questions to try and elicit responses from the participant. They should be used in a conversational manner and only when deemed appropriate.
